# Demonstration of a Robust All-Silicon-Carbide Intracortical Neural Interface

**DOI:** 10.3390/mi9080412

**Published:** 2018-08-18

**Authors:** Evans K. Bernardin, Christopher L. Frewin, Richard Everly, Jawad Ul Hassan, Stephen E. Saddow

**Affiliations:** 1 Department of Biomedical Engineering, University of South Florida, Tampa, FL 33620, USA; ebernardin@mail.usf.edu; 2 Department of Bioengineering, University of Texas at Dallas, Dallas, TX 75080, USA; Christopher.frewin@utdallas.edu; 3 Nanotechnology Research and Education Center @ USF, Tampa, FL 33617, USA; everly@usf.edu; 4 Department of Physics, Chemistry and Biology (IFM), Linköping University, SE-581 83 Linköping, Sweden; jawad.ul-hassan@liu.se; 5 Department of Electrical Engineering, University of South Florida, Tampa, FL 33620, USA

**Keywords:** neural interface, silicon carbide, robust microelectrode

## Abstract

Intracortical neural interfaces (INI) have made impressive progress in recent years but still display questionable long-term reliability. Here, we report on the development and characterization of highly resilient monolithic silicon carbide (SiC) neural devices. SiC is a physically robust, biocompatible, and chemically inert semiconductor. The device support was micromachined from p-type SiC with conductors created from n-type SiC, simultaneously providing electrical isolation through the resulting p-n junction. Electrodes possessed geometric surface area (GSA) varying from 496 to 500 K μm^2^. Electrical characterization showed high-performance p-n diode behavior, with typical turn-on voltages of ~2.3 V and reverse bias leakage below 1 nArms. Current leakage between adjacent electrodes was ~7.5 nArms over a voltage range of −50 V to 50 V. The devices interacted electrochemically with a purely capacitive relationship at frequencies less than 10 kHz. Electrode impedance ranged from 675 ± 130 kΩ (GSA = 496 µm^2^) to 46.5 ± 4.80 kΩ (GSA = 500 K µm^2^). Since the all-SiC devices rely on the integration of only robust and highly compatible SiC material, they offer a promising solution to probe delamination and biological rejection associated with the use of multiple materials used in many current INI devices.

## 1. Introduction

Neuro-engineering is an emerging field which not only seeks to develop engineered therapeutic treatments for a variety of nervous system injuries and disorders, but also looks to understand the functionality of the nervous system. One promising application of neurotechnology is the brain-machine interface (BMI), also known as the brain-computer interface (BCI) [1,2,3,4,5]. Many BCI systems target neuronal electrophysiological signals which interact with signal transducing systems and processing algorithms so they can control external electromechanical devices, such as computers or robotic assistants/limbs [6]. Stimulating BCI systems have modulated the electrochemical environment around neurons to inhibit their activity, as has been demonstrated for Parkinson’s, epilepsy, and pain management; more recently they have been used to replace lost sensory information, as seen with commercial implants like the Cochlear and Argus II [7,8,9]. Using BCI as a bi-directional pathway between electrically active cells and an external device would provide an optimal platform which could utilize closed-loop control, enabling adaptive therapeutic functionality and providing full sensory response for robotic limb replacements.

While many BCI systems operate using noninvasive physiological interfaces, for example, the electroencephalogram (EEG), invasive devices allow intimate contact with cellular populations, thus increasing spatial and temporal resolution as well as signal-to-noise ratio (SNR). The intracortical neural implant (INI) is an emerging technology which targets neurons in the motor cortex, but has also demonstrated applications in the sensory and visual cortex [1,2]. INI can extract high-quality electrophysiological signals, such as action potentials for the control of complicated external mechanical devices [4,10].

Unlike current state-of-the-art implantable neural devices with macro-electrodes, e.g., deep-brain and spinal cord stimulators, cortical INI devices have experienced major issues which have prevented their increased acceptance and use in clinical therapeutic applications. One of the foremost problems is a questionable long-term reliability, which has manifested itself as signal degradation leading to complete loss of device functionality [11,12,13]. The reliability issue has been attributed to both biotic and abiotic sources. While many INI are initially tolerated by the brain after surgical implantation, the inflammatory response progresses in a pattern akin to neural degenerative diseases, silencing nearby neural signals and eventually encapsulating the device in scar tissue [14,15]. Abiotic mechanisms have been attributed to chemical interactions, such as oxidation, leading to corrosion of the device material [16,17,18]. Water absorption has also played a part and has been linked to physical degradation, swelling, delamination, and cracking in multiple-material-layer devices [12,16,17,19,20]. These issues must be addressed with the goal of extending the viability of these devices from only a few years to multiple decades so that they can be sanctioned for widespread use in humans.

One approach to addressing abiotic mechanisms has been the materials required to fabricate the INI [21]. Many state-of-the-art INI devices are constructed using stacks of multiple materials, such as silicon (Si), titanium (Ti), platinum (Pt), parylene C, and polyimide [5,22,23,24]. Not only must each material tolerate the biological environment without exacerbating the inflammatory response, each of the materials used must physically withstand the environment as well as interact well with each other. Our group believes an alternative material strategy may address both issues simultaneously. The device would be constructed exclusively of one material which has a demonstrated track record of physical robustness, chemical inertness, and a great degree of biological tolerance: crystalline silicon carbide (SiC) [25,26,27,28,29,30,31]. Additionally, the amorphous form of SiC (*a*-SiC) has also been demonstrated as an effective insulating coating which does not take up water and is very compatible with neural cells [25,32,33]. 

Here we report on the development, fabrication, and characterization of monolithic SiC microelectrode arrays (MEAs). These arrays were composed completely of one material, SiC. Hexagonal crystalline SiC (4H-SiC) served as both the substrate and conductive electrode elements. Use of alternate polarity SiC, i.e., n- and p-type regions, creates a p-n diode to provide isolation. *a*-SiC served as a conformal, top-side insulator coating to prevent the electrochemical environment from shorting the p-n diode. The novel idea behind an all-SiC device is the concept that a single-material system would be inherently more robust since it does not rely on the heterogeneous integration of multiple dissimilar materials. Another benefit is that SiC uses the same well-established processing techniques developed within the silicon device industry. The result of this study shows that all-SiC microelectrodes interact with an electrochemical environment primarily through capacitive mechanisms with an impedance comparable to gold electrodes. Furthermore, while it cannot deliver charge as efficiently as other conventionally used microelectrode materials, like iridium oxide, the expanded water window of SiC increases the capacitive charge delivery to levels on par with that necessary to evoke physiological activity.

## 2. Materials and Methods

### 2.1. All-SiC Device Fabrication Process

The all-SiC neural devices reported here were developed using standard semiconductor device fabrication processes. SiC epitaxial wafers were grown at Linköping University (Linköping, Sweden) via hot-wall chemical vapor deposition (CVD) [34,35]. A ~5 µm thick, low-doped (aluminum) p-type epitaxial layer of 4H-SiC was homoepitaxially grown on a quarter wafer of 4° off-axis 4H-SiC (0001) [36]. The doping density of the p-type base epitaxial layer was ~1 × 10^16^ cm^−3^. This was followed by the deposition of an opposite polarity 2.5 µm thick, heavily doped (nitrogen) n^+^ film which is known to display semi-metallic conduction [36]. The two layers form a pn junction diode, which provided electrical isolation between adjacent electrode mesa traces and offered minimal leakage current. 

An insulating *a*-SiC film providing conformal surface insulation was deposited using a PlasmaTherm model 730 Plasma-Enhanced Chemical Vapor Deposition (PECVD) system. The plasma field frequency was set to 13.56 MHz, substrate temperature to 250 °C, and pressure to 900 mTorr. Silane (SiH_4_) and Methane (CH_4_) were used as the reactive gas species at flow rates of 360 sccm and 12 sccm, respectively [25]. Argon (Ar) was used as the carrier gas with a flow rate of 500 sccm. Kapton™ tape was placed on a blank Silicon (Si) companion wafer prior to *a*-SiC deposition for thickness measurement of the deposited film using a Dektak D150 profilometer. The scans additionally allowed determination of film stress by scanning the deposited wafer pre and post *a*-SiC deposition and utilizing the Stoney equation [37].

**Figure 1:**
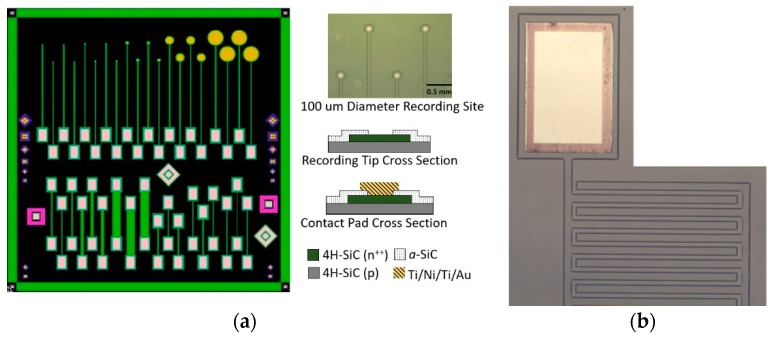
All-SiC planar device used for electrical and electrochemical testing. (**a**) Single-ended electrodes (top) with various recording areas (diameters of 25, 50, 100, 400, and 800 µm) and test structures (bottom) consisting of p-n diodes and resistor mesas of various length and width. An optical micrograph of fabricated all-SiC single-ended electrodes, with 100 µm diameter active recording sites, is provided (top right). Bright areas are windows in the *a*-SiC insulator. (**b**) One end of the all-SiC interdigitated electrode (IDE) showing a metal contact pad (top) and a portion of the two interdigitated 50 µm wide mesas of n^+^ on p base layer with a pitch of 25 µm.

The fabrication sequence to realize the all-SiC microelectrode devices was detailed in Bernardin et al. [38]; however, it has since been refined as explained here. Figure 1a shows the mask design used to produce the single-ended all-SiC electrodes (top) and the test structures (bottom). The as-grown p-n^+^ epiwafers’ surfaces were first functionalized with HMDS (Hexamethyldisilazane Microchemicals GmBH, Ulm, Germany) followed by 15–18 µm of AZ-12XT-20PL positive photoresist (Microchemicals GmBH). The photoresist was patterned by UV exposure (110 mJ/cm^2^) using a Quintel Mask Aligner and developed using the AZ300 developer (Microchemicals GmBH). The thicker photoresist layer than previously reported allowed the Adixen AMS 100 deep reactive ion etcher (DRIE) to etch through 3 µm of the n^+^-doped epilayer, ensuring p base layer exposure and n^+^ conductive trace isolation. The DRIE etched at an approximate rate of 1 µm per minute of SiC with a 0.5:1 mask selectivity. The samples were then placed in the PECVD chamber for the deposition of 200 nm of *a*-SiC as previously described. AZ-12XT (Microchemicals GmBH) was used to define windows in the *a*-SiC film, thus allowing access to the contact pads for device packaging, as well as to expose the active recording sites. The windows were then opened by removing the exposed *a*-SiC layer via reactive ion etching (RIE) in a PlasmaTherm etcher. The process gases used for the RIE steps were 37 sccm of CF_4_ and 13 sccm of O_2_. The process ran for 210 s, with power set to 200 W and pressure at 50 mTorr. The formed window recesses were ~210 nm deep as measured via stylus profilometry.

High-quality ohmic contacts are necessary to transfer signals to and from the semiconductor and the external circuitry [36]. Therefore, the process to create metal contact pads which allow connection to commercial connectors has been optimized to improve device electrical connectivity. The metal lift-off process was defined using a two-layer photoresist step as follows: A 2 µm lift-off resist (LOR10B MicroChem Corp., Westborough, MA, USA), followed by 5 µm of AZ-12XT-20PL, a positive resist. Once exposed to UV and developed, a thin film of titanium (Ti), followed by Nickel (Ni), was deposited in sequence via RF sputtering without breaking vacuum. Liftoff was performed in a Microposit™ Remover 1165 bath. The contacts were then annealed in a rapid thermal processor (RTP) at 1000 °C for 30 s to form a Ti/Ni silicide. A secondary metal lift-off process was performed using Ti and Gold (Au) deposited onto the annealed Ti/Ni stack. The final stack was annealed at 450 °C for 30 min and ohmic contact confirmed via IV measurements. 

### 2.2. All-SiC Evaluation Devices

Two structures were fabricated to characterize the electrical capabilities of the all-SiC devices. In Bernardin et al. [38], all of the electrodes tested were of a single size with a 25 µm recording tip diameter, but varied in lead length from 4 to 10 mm. While we established that trace length did not significantly affect the overall electrical impedance, it has been shown that the geometrical surface area (GSA) of an electrode can be an important factor in overall performance [24]. Therefore, 20 single-ended planar electrodes were constructed with different recording tip diameters of 25, 50, 100, 400, and 800 µm to extract performance as a function of GSA, as shown in Figure 1a. Four (4) planar electrodes of each GSA were fabricated per die. All electrodes had the same lead wire (i.e., the n^+^-doped mesa) length of 3.5 mm and were ~3 µm thick and 50 µm wide.

An important consideration for any electrical device is signal leakage and cross-talk between traces. While many of the INI devices use an insulating material as the basis for metal trace isolation, the semiconductor properties of our single crystal material allow the use of p-n junction isolation to serve this function. Furthermore, the surface coating of *a-*SiC prevents the p-doped substrate and n-doped trace regions from being shorted together when immersed in electrolytic media. To evaluate the efficacy of this insulation strategy, interdigitated electrode (IDE) devices were fabricated to evaluate the insulating properties and reliability of the *a*-SiC coating and p-n isolation between adjacent traces. The IDE devices consisted of two electrodes which were 50 µm wide and 3 µm high. Each of the electrodes contained 22 digits with a length of 1 mm and were spaced 100 µm away from their adjacent digit. Both electrodes were fitted within each other at a spacing (pitch) of 25 µm, with a total enclosed interaction length between both electrodes of 4.5 cm.

### 2.3. SiC Doping and PN Isolation Evaluation

The doping density of the conductive electrode layer was calculated through the characterization of a Schottky diode using the method described by Schroder [39]. Briefly, capacitance/voltage (C/V) and current/voltage measurements (IV) were used on the epitaxial stack prior to device fabrication. An LEI 2017b Mercury (Hg) Probe (Lehighton Electronics, Inc., Lehighton, PA, USA) created a Schottky junction when the Hg came into contact with the top n^+^ epitaxial layer of the wafer. The Hg probe is equipped with a Schottky dot diameter of 0.64 mm and a 3.81 mm diameter Hg return contact. IV measurements were initially performed, sweeping the voltage from −5 V to 5 V at a rate of 0.5 V/s with a Keithley 2400 SourceMeter (Tektronix, Inc., Beaverton, OR, USA) to extract the forward bias turn-on voltage of the Schottky diode. C/V measurements were then made using a Keithley 590 CV Analyzer (Tektronix, Inc.) The C/V measurements were performed by sweeping the voltage 1 V under the reverse bias of the Schottky junction. Measurements were taken at a frequency of 1 MHz and at a rate of 150 mV/s. The C/V measurements were then used to calculate doping density.

As previously described, p-n junctions formed during 4H-SiC epitaxial growth were used to provide electrical isolation between electrodes. PN diodes characteristically pass current in only one direction after a potential is provided, known as the turn-on voltage, while they resist current flow in the opposite direction until a second potential is achieved, known as the reverse breakdown voltage. IV measurements were utilized to establish the turn-on potential, characteristic reverse leakage current, and reverse breakdown voltage. PN diode test devices were included on the all-SiC single-ended electrode die (Figure 1a bottom) consisting of a square, n-doped epilayer mesa with a metal contact which was surrounded by a second metal contact to the p-doped base epilayer separated by 10 µm. The Keithley 2400 SourceMeter was used to facilitate IV measurements from potential limits of −20 V to 5 V at a rate of 0.5 V/s. Turn-on voltage was estimated by plotting current vs voltage on a semi-logarithmic current scale, and extrapolating the linear forward current to where it intersects the voltage axis. The reverse breakdown voltage occurs at the point where the diode’s current increases rapidly with the applied reverse voltage and we used 1 nA as our quantitative measure of reverse breakdown voltage for comparative purposes. The reverse leakage current was estimated from the root mean square of the current between the turn-on and breakdown potentials.

The IDE devices (Figure 1b) were evaluated to examine the isolation between distinct traces. In this case, complete isolation consisted of two back-to-back p-n junction diodes, both requiring large biases to surpass. The IDEs were characterized using the Keithley 2400 SourceMeter. The source contact was connected to one of the two outer electrodes of the IDE, and the return contact was connected to the center, or common, electrode. IV measurements were performed from potential limits of −50 to 50 V at a rate of 5 V/s. Device failure was marked using the same criteria as used for the junction diode evaluation (i.e., when the current exceeded 1 nA). We tested a total of 10 IDE structures. 

### 2.4. Electrochemical Characterization of 4H-SiC Material

All electrochemical evaluations were performed in phosphate-buffered solution (PBS) of composition 137 mMol NaCl, 27 mMol KCl, 100 mMol Na_2_HPO_4_, and 17.6 nMol KH_2_PO_4_. The pH was titrated using HCl to reach 7.4 pH. All measurements were taken at laboratory temperature of ~22 °C. A model 604E Series Electrochemical Analyzer/ Workstation (CH Instruments Inc., Austin, TX, USA) and vendor-provided software were used to characterize the 4H-SiC electrodes using cyclic voltammetry (CV) and electrochemical impedance spectroscopy (EIS). A three-electrode system was used consisting of a 127 µm diameter, ~5 cm wide Pt wire (A-M Systems, Sequim, WA, USA), a 5 cm long Ag|AgCl reference electrode of 254 µm diameter, and the all-SiC electrode working electrode.

The EIS and CV measurements were performed on four (4) die containing 20 electrodes of five different sizes (N = 16 for each electrode size). EIS measurements consisted of the application of a 10-mV root mean square (rms) sinusoidal wave at a frequency range between 0.1 Hz and 100 KHz, and recording of the returned current response 12 times per decade. A circuit model representation for the 4H-SiC electrochemical interface was selected from [40,41] as a basis for interpreting electrochemical impedance spectroscopy data. This model was used in ZSimpWin software (Amtek Scientific Instruments Inc., Austin, TX, USA) in an iteration mode to calculate values for the circuit components.

The initial CV measurement started at the open circuit potential, swept to the negative potential of −0.6 V at a rate of 50 mV/s, reversed direction to the positive potential of 0.8 V, and returned to the negative potential, repeating the cycle three times. The potential limits of −0.6 to 0.8 V, which correspond to the limits reported for Pt, were chosen to allow direct comparison with previously published results [24,42]. A second CV measurement was performed using the same boundary conditions, but the potential limit was widened 0.1 V after each complete scan. Once it was noticed that the onset of a large current occurred, which was a typical marker of water electrolysis, that potential became that electrode’s extended limit. The sweeps were performed until both oxidation and reduction potential limits were established, after which the final three CV sweeps were performed.

## 3. Results

### 3.1. Device Characterization

**Figure 2:**
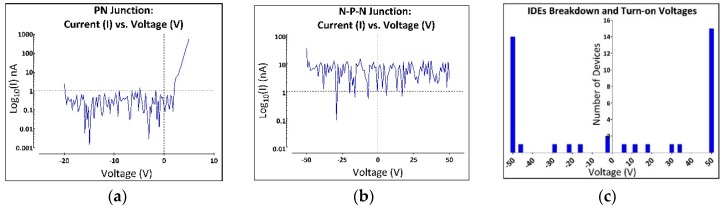
All-SiC device electrical characterization. The electrical properties of the device were first tested by performing current vs voltage (IV) measurements on the fabricated test structures. (**a**) IV measurements were performed over a range of −20 V to 5 V on the p-n diodes post fabrication. The results, shown on a semi-log plot, demonstrate a typical forward-biased turn-on voltage of ~2.3 V and a leakage current well below 1 nA out to −20 V. (**b**) Conductive trace mesa isolation measurements via IV sweeping from −50 to 50 V in air. Note that the long p-n junctions provided excellent isolation with a slight current leakage of less than 7.5 nA_rms_ over the measured voltage range. (**c**) Histogram from the insulation/isolation measurements performed on 20 IDE structures of Figure 1b. IV characterization for 20 IDE devices were taken from −50 V to 50 V. Of the 20 devices, only 2 showed significant current flow at the p-n junction’s turn-on voltage (−2.3 V), while 4 IDEs showed current flow at cathodic potentials between −16 V to −50 V and the remaining 14 continued to block to at least −50 V. Under reverse bias conditions, 5 IDEs experienced leakage current between 6 V to 35 V, while the remaining 15 continued to block up to 50 V.

Capacitance versus voltage measurements of Schottky contacts to the n^+^ layer verified that the doping on the n^+^ epi layer was on the order of 3.45 × 10^18^ cm^−3^ which was consistent with our epi-doping estimates of ~3 × 10^18^ cm^−3^ from prior growth runs using the same level of intentional nitrogen doping [38]. Typical doping of the p-type base epitaxial layer was ~1 × 10^16^ cm^−3^. Figure 2a displays a semi-log IV plot from a selected p-n junction diode. The p-n diode measurements showed a high-performing p-n diode with a typical turn-on voltage of ~2.3 V and a reverse bias leakage that fell below our equipment limit of less than 1 nA. The turn-on voltage value is comparable to values reported in the literature [36,43,44].

The n-p-n junction, which isolates adjacent leads through the substrate, was also tested by repeating the IV measurements using the contact pad of one device to the contact pad of its neighboring device. This test helped demonstrate the effectiveness of the n-p-n junction in isolating individual electrodes (and devices) from one another. These measurements were performed under dry (air) conditions at a voltage range of −50 to 50 V. Figure 2b shows that the n-p-n diode has a slight current leakage of less than 7.5 nA_rms_ over the measured voltage range. 

Of the 20 IDE devices tested (Figure 2c), only 2 demonstrated a significant current flow at the turn-on potential for the junction diodes (2.3 V) and 4 IDEs showed current flow at cathodic potentials between −16 V to −50 V. The remaining 14 forward-biased electrodes continued to block currents to at least −50. Under reverse bias, 5 IDEs experienced leakage currents of more than 1 nA between 6 V to 35 V, while the remaining 15 continued to block up to 50 V.

### 3.2. Electrochemical Characterization

**Figure 3:**
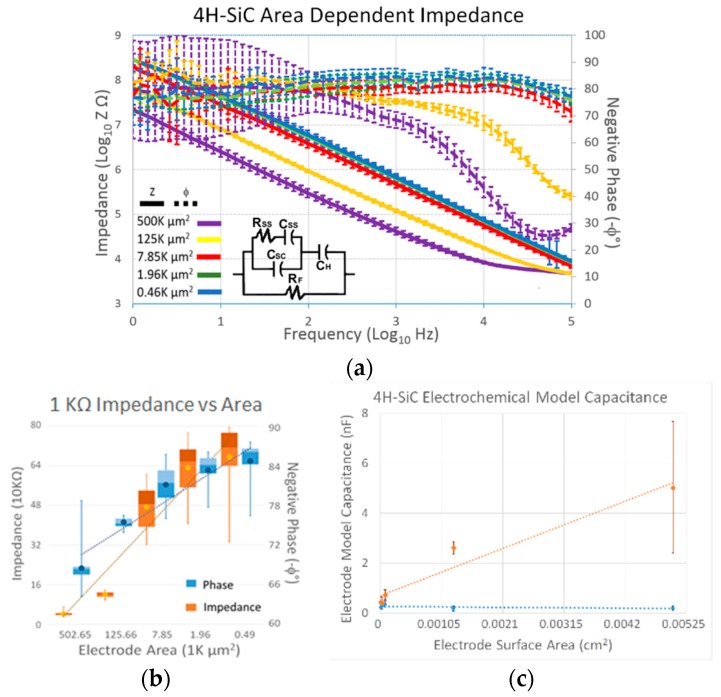
(**a**) Electrochemical impedance spectroscopy (EIS) performed on 4H-SiC electrodes of varying geometrical surface areas (GSA). The error bars represent the standard deviation. The inset shows the basic circuit model which produced an impedance profile which was similar to that which was obtained in experimental investigation. (**b**) A box and whiskers plot for the impedance and phase of the 4H-SiC electrodes at the frequency of 1 kHz across the 5 tested areas. The mean impedance and phase is represented with a dot. (**c**) The value of the space charge capacitance (CSC) obtained through the electrochemical modeling of the EIS for the 4H-SiC electrodes divided by the geometric surface area of each electrode. All tests were performed in PBS that was naturally aerated (i.e., no N_2_/Ar gas bubbling to remove dissolved O_2_) and possessed a pH of 7.4.

The results from the EIS performed on the 4H-SiC electrodes in 7.4 pH aerated PBS are shown in Figure 3a. The electrode interaction with the electrolyte displayed a nearly purely capacitive property at frequencies less than 10 kHz, as is displayed by the phase angle of ~80 °C for the electrodes with less than 7.85 K µm^2^ geometrical surface area. Another notable trend was that the impedance decreased with an increase in electrode area. Figure 3b displays the impedance and phase obtained from the electrodes when measured at 1 kHz, the frequency associated with the action potential associated with cortical neural processes. The smallest electrode (496 µm^2^) had an average impedance of 675 ± 130 kΩ, which decreased to 46.5 ± 4.80 kΩ for the largest, 500 K µm^2^ area electrodes. The trend lines added to the graph show a relative linearity associated with all the electrodes.

The inset in Figure 3a shows the circuit model which was used in the ZSimpWin software to predict the impedance response similar to the data obtained from our actual electrodes. The circuit model consists of the Helmholtz double-layer capacitance (C_H_) and Faradaic resistance (R_F_) normally seen in a Randles circuit model, but includes an additional component exclusive to semiconductors [40,41]. The additional component models the capacitance of the space charge region (C) and the accompanying charging component corresponding to the speed of the change in surface state (R_SS_ and C_SS_). These components are directly derived from the metal/semiconductor junction, where the interaction of the electrolyte and semiconductor leads to the fixation of the Fermi level and a bending of the conduction and valence energy bands which, in turn, drive the semiconductor surface into depletion. The Faradaic impedance (R_F_) started near 1 GΩ for the largest electrode, increasing to approximately 6 GΩ for the smallest electrodes. The Helmholtz double capacitance (C_H_) was on the order of 3 nC for the large-scale electrodes and approached 0.5 nC for the small electrodes. The surface state resistance (R_SS_) was in the hundreds of kΩ with the smallest electrodes and was only in the tens of kΩ for the largest electrodes.

**Figure 4:**
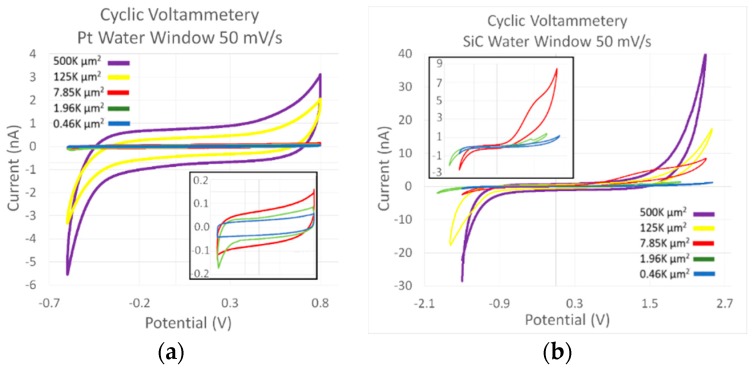
(**a**) CV plot showing a single curve from the cyclic voltammetry evaluation of a select 4H-SiC electrode. The curves were bounded at the potentials typically used for platinum or iridium electrodes of −0.6 V and 0.8 V. The inset shows detail for the three smallest electrodes. (**b**) A plot of the second CV curve from a select electrode. The ramp rate was the same as for (**a**), but the potential limits were extended to the onset of a large current flux. All tests were performed in PBS that was naturally aerated (i.e., no application of gas bubbling) and possessed a pH of 7.4.

Figure 4a displays select representative CV curves for each of the electrodes reported in this study taken across the potential water limit for Pt. Figure 4a, on the other hand, shows select curves using the extended water window from the all-SiC electrodes. Both micrographs demonstrate a decrease in the hysteresis cycle when the area is decreased. Once again, CV data supports a dominant capacitive interaction mechanism with the electrolytic ions. The current remains constant for the CV sweep, until it nears the potential limit on either side of the test. At this point, the charge potential is reversed, leading to a large influx of current which follows the resistor-capacitor (RC) charging process. After the charging, the current once again remains constant until the next potential ramp shift. While many of the devices produced curves that were strictly capacitive, a few of the electrodes show increased Faradaic oxidation currents. These oxidation reactions were noticed exclusively on the tests where the potential limits were being evaluated, and an example can be seen in Figure 4b on the 7.85 K µm^2^ area electrode. A second observation was that the coupled reduction current was not present on the same electrodes. While these electrodes posed an electrochemical difference, the overall interaction was relatively consistent. This is seen in the variability lower than 5% for the potential limits. The cathodic potential limit was observed to be −1.98 ± 0.08 V while the anodic limit was 2.77 ± 0.07 V.

The difference between the electrodes is more apparent when evaluating the overall charge storage capacity (CSC) and charge delivered per phase. Table 1 displays the mean and standard deviation of the mean for all the tested electrodes. The electrodes once again demonstrate a simple area relationship. The smaller electrodes have the largest values for CSC, which decreases with the increase in area. The largest electrodes deliver the largest amount of charge per phase, which decreases with a decrease in area. 

## 4. Discussion

Both the neuroscience and biomedical communities have been focused on improving the overall reliability of implantable microelectrode systems. Complications have been demonstrated to arise from both biotic and abiotic mechanisms. Biotic systems have been connected to the inflammatory response, which has been linked to material modulus mismatch and foreign body response, resulting in a tissue response on par with neural degeneration [45,46,47]. Material degeneration, oxidation, water absorption, swelling, stress fractures, and delamination have all been associated with abiotic mechanisms [12,20,48,49,50].

For many implantable neural interface (INI) devices, the electrical interaction with the physiological environment has been an electrode. While electrodes are in their simplest form devices composed of at least one conductive material and one insulating material, overall device reliability remains complicated. The fact that the device needs to be fabricated from at least two materials raises concern on dissimilar material surface interaction in order to minimize delamination [12,51,52,53]. The materials are constantly exposed to a harsh, oxidizing environment with large variation in pH, which has required a careful evaluation of chemical resistivity to avoid degeneration and production of chemical species which are toxic or exacerbation of the inflammatory response [45,54]. Another consideration has been the characterization of the electrochemical interface, where Faradaic reactions have required careful consideration to avoid the generation of harmful reactive species and hydrogen/oxygen gasses [55,56]. Finally, a recent focus has been on material hardness and flexibility as there has been evidence showing that a mismatch between the mechanics of the material and soft tissues which are in constant micromotion has generated additional biological damage [47,48,57]. For clarity it should be noted that the all-SiC concept presented here does not address the micromotion issue.

While conductors and insulators are normally the materials of choice for electrode fabrication, another class of materials, semiconductors, offers an interesting fabrication methodology that allows electrode construction from a single-material system. The advantage of this method is that delamination is highly improbable. Semiconductors, unlike conductors, require a certain amount of applied potential to promote electrons from the valence band, over the forbidden energy band gap and into the conduction band where they can move freely; however, while this energy level is much smaller than that required for insulators, it still is much larger than the potential found in neuronal signals. This issue has been solved by the fact that semiconductors have been modified into a much more conductive state through the addition of atoms into their crystalline matrix, referred to as dopants. Unlike traditional metallic conductors, these dopant atoms enable conduction through either the donation of an electron into the conduction band of the semiconductor, thus producing n-type “electron-rich” material, or by accepting valence band electrons, thus creating “holes” to create a p-type, or positively charged, semiconductor. By switching the dopant type (donors for n-type or acceptors for p-type) during semiconductor growth, a junction is created between the n- and p-type materials. At this PN junction, electrons from the n side diffuse to the p side, while holes from the p side of the junction diffuse in the opposite direction. This creates a region devoid of mobile carriers which then blocks the flow of further mobile charge carriers. This rectifying junction thus blocks current flow and only through the application of a bias potential, called forward bias, may appreciable diffusion current flow again. In a silicon PN diode this potential is ~0.7 V, whereas in 4H-SiC it is ~2.3 V. Thus, a wide-bandgap semiconductor is advantageous since the diode remains in its off state at considerably higher potentials. 

Semiconductor junction electrode isolation was demonstrated in the Utah intracortical array, now known as the Blackrock microelectrode array [58,59]. Although the feasibility of the method was demonstrated, p-dopant thermo-migration shorted multiple electrodes together and a large surface contamination required silicon dioxide surface passivation. One important factor which was mentioned concerned stimulation. At voltages above 0.7 V, the silicon junction forward turn-on voltage, the diode turned on and injected current into the substrate, thus shorting all of the electrodes together. Later reports have shown that silicon and its related material derivatives (SiO_2_ and Si_3_N_4_) have been connected to chronic neuroinflammation, most likely due to chemical reactivity and physical modulus mismatch [11,12,13,60]. Many other semiconductor materials demonstrate traits that would not allow their use in reliable INIs, such as gallium arsenide, and have demonstrated biological toxicity [61]. Diamond and gallium nitride have demonstrated elements of anodic oxidation and subsequent material corrosion [62,63]. Boron nitride has demonstrated an extremely low electron mobility, leading to increased resistivity and signal attenuation [64]. While these issues from many semiconductor materials limit their ability for use in INI applications, one semiconductor material, silicon carbide (SiC), appears ideally suited for reasons which will now be explained.

Silicon carbide is a semiconductor which possesses extreme resistance to corrosive chemistries, experiences only limited oxidation, and has demonstrated no appreciable toxicity [65,66,67,68]. We have demonstrated that hexagonal silicon carbide junction isolation electrode devices have a much higher forward bias turn-on potential than Si (2.3 V vs. 0.7 V). The Utah microelectrode array was driven into forward bias conditions during the application of anodic potential stimulation, at levels of nominally ~0.8 V, which ensures that the device remains within the safe water window for Pt. While our device used a similar junction isolation technique, there are major differences which enhanced our successful application of a single-material electrode. First, our electrodes are composed of heavily doped n-type 4H-SiC, while our substrate is lightly doped p-type 4H-SiC, which forms the junction isolation bias. The 4H-SiC device isolation would actively pass minority carrier current into the substrate with cathodic stimulation greater than −2.3 V, which thus represents the upper limit in applied bias. This junction configuration also required the application of potentials well in excess of 50 V to bias the junction into reverse breakdown. While 5 electrodes (25%) demonstrated a failure to sufficiently block reverse potentials between 6 V and 50 V and demonstrated significant breakdown current, this failure was well above the anodic water window of 2.8 V. Therefore, we conclude the possibility of device failure due to anodic bias to be very small.

It was noticed that the devices were not functioning in the same way as reported in the Utah investigation once the turn-on potential was reached. Application of cathodic potential to the IDE electrodes revealed that only two (2) demonstrated a significant current flow at potentials above −2.3 V, the turn-on potential for the junction diodes. Only 4 more electrodes showed current flow, and that happened at cathodic potentials between −16 V and −50 V. The remaining 14 forward-biased electrodes continued to block current to at least −50 V. At forward junction potential, the current was offered two parallel pathways. One was a large surface area, essentially the entire 2-dimensional contact area enclosed within the electrode and the trace added together, with a very short length that connected to the entire device surface area of the n-type substrate. The second path consisted of a surface area composed of the 5 µm thick p-type layer and the length of one side of the electrode (1 mm). The spacing between the electrodes was 100 µm. The substrate provides a good sink (<1 Ω resistance) which is more favorable than the pathway to the neighboring adjacent electrodes (>10 KΩ resistivity). Of course, the further away the electrodes are, the greater the signal attenuation. Unfortunately, one issue exists with injecting current into the substrate in that it is potentially in contact with the electrochemical environment. The substrate would have to be totally encapsulated in *a*-SiC to safely operate (i.e., the backside and edge of the device would need to be coated with *a*-SiC). 

It should be noted that while our device is constructed from a single material, SiC, it was not a single piece of material. *a-*SiC has demonstrated excellent insulating capability, both in vitro and in vivo, and does not allow diffusion of metal ions (Na, K, Fe) as has been observed with SiO_2_ [33,69]. The *a*-SiC coating that we chose was extremely thin, at ~200 nm, increasing the chance to reduce its insulating properties, which would potentially short both sides of the junction diode together. A thicker insulating film can be used in next-generation devices. We have shown that the pn junctions provide excellent substrate isolation, but this single junction isolation showed issues 20–30% of the time. The solution to both issues would be to bury the n^+^ conductor layer under a p-type epitaxial layer and then form an n^+^ conductive via through the p-type film to the device surface by use of ion implantation, which is a common process step in SiC power electronic devices. An added benefit of this increase in complexity would be that this could add active negative bias on the innermost layer, thus ensuring a counter bias against an active junction [70].

Many commonly used electrode materials use dual mechanisms to exchange charge within an electrochemical environment. These materials have a Faradaic element, wherein the charge is transferred through a surface oxidation or reduction reaction [71,72,73]. Materials that rely heavily on this charge transfer mechanism are the noble metals and iridium oxide (IrOx). The secondary method is through capacitive means, normally due to the absorption of water—which is an excellent insulator—across the surface of the electrode; this essentially leads to a separation of the charged ions in the electrolyte and electrons in the electrode, giving rise to a parallel plate capacitance [74]. Titanium nitride (TiN) and carbon materials have been associated with this capacitive mechanism to transfer charge [75,76,77]. With materials that primarily rely on capacitive charge transfer, large surface areas are desired to achieve increased charge transfer.

Five all-SiC electrodes of increasing geometrical surface area, via doubling electrode diameter from 25 to 800 µm (200 µm was left out to save real estate), were characterized electrochemically in PBS. Our previous investigation evaluated only 25 µm diameter electrodes and demonstrated capacitive interaction with little Faradaic activity [38]. That study demonstrated that 4H-SiC fits the same electrochemical model as reported for semiconductor electrodes [41,78]. 4H-SiC is extremely chemically resistant, only possessing limited surface oxidation for Faradaic chemical electron transfer, thereby increasing overall impedance and resulting in a reduced current transfer path. Instead, SiC demonstrated charge transfer through a parallel capacitive pathway. Unlike metallic conductors which rely mainly on double-layer capacitance for charge transfer, semiconductors possess an additional capacitive element. Charged ions on the surface of the semiconductor form a Schottky barrier, where the Fermi energy level equalizes, resulting in bending of the valence and conduction bands. Just as with the junction diode, majority carriers deplete through electron-hole annihilation, resulting in the presence of a space charge region [41,78]. This space charge region creates a large distance between the semiconductor bulk charge and the ions on the surface when compared with the double layer, leading to much smaller capacitances. At levels of doping below 10^19^ cm^−3^, the space charge capacitance, which is in series with the double-layer capacitance, dominates the overall electrode capacitance [41,78].

Electrochemical characterization allowed us to evaluate the electrical functionality for the various 4H-SiC electrodes with respect to area. The electrochemical impedance of 4H-SiC electrodes versus geometric surface area, obtained at a frequency of 1 kHz, has been indicated in the box plots displayed in Figure 3b. The semiconductor electrochemical circuit model described earlier was used to calculate the resistive and capacitive elements for the electrodes, and the space charge capacitance and double-layer capacitance are displayed in Figure 3c. The overall resistance of the electrodes, modeling the electron exchange through chemical interaction between the electrode surface and the ionic media, increases exponentially with increasing surface area. This finding is in line with chemical reactions which are surface limited.

The differences between the two capacitive elements show that our devices possessed irregularities that need to be investigated. While the double-layer capacitance showed that an increase in capacitance accompanied increasing area, the capacitance associated with the space charge, or depletion, region of the semiconductor does not show an appreciable increase with surface area but stays nearly constant. Close examination of the electrodes with 7.85 K µm^2^ or less showed an increasing capacitance with increasing surface area, while the electrodes of 125 K µm^2^ or greater showed an inverse relationship. The static capacitance has also been displayed through individual CV micrographs where Figure 4a showed an increasing current saturation for each electrode size, from smallest to largest. However, Figure 4b shows that electrodes larger than 125 K µm^2^ surface area possess nearly the same level of current saturation throughout the potential ramp. If the Helmholtz double-layer capacitance increases with surface area, and the potential rate remains the same, the lack of increased current associated with increased electrode diameter could be attributed to an issue with the space charge capacitance (C_SC_). Research has indicated that increasing Schottky interface area does not change depletion depth, but instead leads to increased effects due to edge effects [79,80,81]. Additionally, while fringing effects usually increase the effective capacitance, resistive shunts are formed at the edge of Schottky barriers which effectively reduce current [81]. It should also be noted that decreased capacitance has been reported due to crystalline defects [82]. Epitaxial growth defects known as carrots (or comet trails) defects, island growth, and step bunching have been directly associated with Schottky rectification [83]. Although reported defect densities are low for 4H-SiC (10–20 per cm^2^), our large-area electrodes increase the chance of containing at least one defect per device. To overcome some of these issues, double-trench isolation, consisting of multiple layers of alternating p- and n-type material, along with the addition of semi-insulating or intrinsic layers around the devices could provide additional leakage isolation and guard rings to counter edge effects [70]. As with all solutions, the process would require additional device complexity, with additional epitaxial growth stages, masking, and processing stages. However, it would also allow one additional benefit in that the double-trenched electrodes would not require an external *a-*SiC coating, making the device truly monolithic, composed of a single crystalline material, eliminating the possibility of insulation delamination altogether.

Microelectrode neural implantation devices normally have electrodes with areas in the thousands of µm^2^, or even smaller. Issues were discovered with the 4H-SiC electrodes with geometric area larger than 8000 µm^2^ used as comparison when evaluating the capabilities of these electrodes concerning cathodic charge storage capacity (CSC_c_). The CSC_c_ for 4H-SiC electrodes with a potential limit of −0.6 V was 64 µC/cm^2^ for electrodes of 7854 µm^2^ area, increasing to 194 µC/cm^2^ for 490 µm^2^. Increasing the potential window to −2.0 V, the CSC_c_ was 1.51 mC/cm^2^ for an area of 7854 µm^2^ and 5.53 mC/cm^2^ for a 490 µm^2^ area. Comparatively, these electrodes demonstrate charge storage comparable to that reported for Pt electrodes (0.8–1.6 mC/cm^2^ for 6500 µm^2^) [84], and only slightly lower than that reported for TiN (2.47 mC/cm^2^ for 4000 µm^2^) [85]. 4H-SiC has demonstrated an electrochemical interaction dominated through capacitive electron transfer, much like TiN. Materials like poly(3,4-ethylenedioxythiophene) (PEDOT) (11.4 mC/cm^2^ for 4000 µm^2^) [86], activated iridium oxide (AIROF) (24.0 mC/cm^2^ for 1000 µm^2^) [87], and electrodeposited iridium oxide (EIROF) (48.7 mC/cm^2^ for 385 µm^2^) [33] achieve electron transfer through Faradaic chemical reaction mechanisms, leading to larger CSCc values exceeding capacitive 4H-SiC by at least a factor of 10.

However, there are many improvements that can be made to put SiC on a slightly better stimulation footing. The first is to increase the doping density. A boron-doped diamond coating (2 × 10^21^ cm^−3^) was used on a 5000 µm^2^ area electrode with a CSC_c_ of 10 mC/cm^2^, which is comparable to the value reported for PEDOT [88]. The increased charge storage required multiple improvements, but 4H-SiC can use the same methods. Like 4H-SiC, the diamond required an extended potential of −2 to 1.5 V to increase its capacitive charge delivery. An increase in the doping levels of 4H-SiC to at least 10^20^ cm^−3^ will decrease the resistance of the electrode through an increase of majority charge carriers. The hole mobility of boron p-type doping in diamond was 50 cm^2^/Vs at 300 K for 10^21^ cm^−3^ [89]. Achieving a doping level of 10^20^ cm^−3^, an order of magnitude lower, would be more realistic for 4H-SiC as it requires ion implantation and subsequent dopant activation to achieve levels of doping above 10^19^ cm^−3^. The electron mobility for n-type 4H-SiC would be comparable to the reported level for diamond, around 30 to 50 cm^2^/Vs [90]. The diamond reported in [88] was highly nanostructured, producing a large increase in surface area. The SiC electrodes reported here were relatively flat. Many methods have been reported that can increase the surface area of SiC, like the formation of surface nanostructures or creating a more porous surface [91,92,93]. However, as we have demonstrated with our large-area electrodes, increasing the surface area of SiC may not produce a straightforward increase in electrochemical interaction and would require more investigation.

We have demonstrated a 4H-SiC electrode composed of single crystal, n-type semi-metallic material grown on a p-type base and reported on the suitability of this material system for biological utilization. Here we show that 4H-SiC interacts within an electrochemical environment primarily through a capacitive mechanism, dominated by a Schottky depletion capacitance instead of the classic double-layer capacitance. Additionally, the two-layer doping junction provided excellent, low-leakage isolation through the substrate, superior to that demonstrated by silicon devices. It is interesting to note that while PN junction isolation did perform as expected, there is the possibility of AC signal coupling across the junction for large signal amplitudes if the junction is not DC biased into reverse bias. Since we are able to control the potential between the p base layer and the n^+^ semi-metallic layer (and, thus, the electrochemical environment) such that the PN diodes are in reverse bias, we do not anticipate that this will be an issue, but it certainly is an important issue that must be accounted for during device operation. Finally, the impedance of the material is comparable to those of many of the noble metals, like gold and platinum, but the charge storage capacity was lower than those of commonly used materials like iridium oxide and PEDOT. The electrodes have demonstrated adequate functionality and should be able to interact within the physiological environment and interface with electrically active tissue and cells.

## 5. Conclusions

A monolithic all-SiC MEA has been fabricated, where 4H-SiC served as the base (substrate), as well as the conducting traces (electrodes) of the device, while *a*-SiC was used as the insulating layer. Conventional silicon photolithographic processing techniques where employed in the design and fabrication of the all-SiC device. Electrical testing of the p-n^+^ junction demonstrated that the 4H-SiC device is capable of blocking a forward-biased voltage up to 2.3 V and a reverse voltage of more than 10 V. Furthermore, electrochemical results show that the 4H-SiC microelectrodes interact with an electrochemical environment primarily through capacitive mechanisms and have impedance comparable to that of gold electrodes. However, the 4H-SiC devices cannot deliver charge as efficiently as other conventionally used microelectrode materials, such as iridium oxide. Using the already established silicon processing techniques, a variety of different forms of monolithic SiC neural devices are possible such as the single- or multiple-shank planar neural probes. All studies and data collected thus far indicate that the monolithic SiC neural device can aid in the advancement of chronic INI use in clinical settings. However, to demonstrate a fully functional INI device, further studies must be performed. Future work includes conducting accelerated aging experiments with our all-SiC device to test the performance and stability of the insulating *a*-SiC film. This future study will evaluate whether our insulator adheres to the all-SiC device or delaminates under physiological conditions. In addition, extensive in vivo studies will be conducted to determine the extent to which the neural probes will function post implant.

## Figures and Tables

**Table 1:** Cathodic and anodic charge storage capacity and charge per phase *.

	Cathodic				Anodic			
**Electrode Area (µm^2^)**	**Charge Storage Capacity (mC/cm^2^)**		**Charge Per Phase (nC)**		**Charge Storage Capacity (mC/cm^2^)**		**Charge Per Phase (nC)**	
	−0.6 to 0.8 V	−2.0 to 2.8 V	−0.6 to 0.8 V	−2.0 to 2.8 V	−0.6 to 0.8 V	−2.0 to 2.8 V	−0.6 to 0.8 V	−2.0 to 2.8 V
502,651	4.38 × 10^−3^ (1.24 × 10^−4^)	8.00 × 10^−2^ (0.644)	22 (0.625)	380 (0.642)	3.70 × 10^−3^ (4.81 × 10^−5^)	0.257 (0.52)	18.4 (0.242)	1340 (0.520)
125,663	1.18 × 10^−2^ (1.42 × 10^−3^)	8.61 × 10^−2^ (1.60 × 10^−2^)	14.8 (1.78)	108 (20.1)	7.02 × 10^−3^ (2.9 × 10^−4^)	0.115 (2.28 × 10^−2^)	8.82 (0.369)	144 (28.7)
7854	2.98 × 10^−2^ (2.60 × 10^−3^)	1.01 (0.185)	2.34 (0.204)	79.5 (14.5)	6.22 × 10^−2^ (3.10 × 10^−3^)	1.29 (0.113)	4.89 (0.244)	102 (8.87)
1964	6.46 × 10^−2^ (2.07 × 10^−3^)	1.51 (0.167)	1.27 (4.06 × 10^−2^)	29.6 (3.27)	0.177 (9.22 × 10^−3^)	1.37 (0.187)	3.48 (0.181)	27 (3.66)
491	0.194 (5.67 × 10^−3^)	5.53 (1.38)	0.953 (2.78 × 10^−2^)	30.4 (6.63)	0.406 (2.22 × 10^−2^)	5.42 (0.666)	2.00 (0.109)	41.7 (3.81)

* Data displayed for both the standard Pt water window as well as for the water window empirically derived for SiC. Standard deviation of the mean is shown in parentheses.

## References

[1] Donoghue J.P. (2008). Bridging the brain to the world: A perspective on neural interface systems. Neuron.

[2] Lebedev M.A., Nicolelis M.A.L. (2006). Brain-machine interfaces: Past, present and future. Trends Neurosci..

[3] Wolpaw J.R., Birbaumer N., McFarland D.J., Pfurtscheller G., Vaughan T.M. (2002). Brain-computer interfaces for communication and control. Clin. Neurophysiol..

[4] Lobel D.A., Lee K.H. (2014). Brain machine interface and limb reanimation technologies: Restoring function after spinal cord injury through development of a bypass system. Mayo Clin. Proc..

[5] Maynard E.M., Nordhausen C.T., Normann R.A. (1997). The Utah intracortical electrode array: A recording structure for potential brain-computer interfaces. Electroencephalogr. Clin. Neurophysiol..

[6] Jorfi M., Skousen J.L., Weder C., Capadona J.R. (2015). Progress towards biocompatible intracortical microelectrodes for neural interfacing applications. J. Neural Eng..

[7] Luo Y.H.-L., da Cruz L. (2016). The argus^®^ II retinal prosthesis system. Prog. Retin. Eye Res..

[8] Markowitz M., Rankin M., Mongy M., Patino B.E., Manusow J., Devenyi R.G., Markowitz S.N. (2018). Rehabilitation of lost functional vision with the argus ii retinal prosthesis. Can. J. Ophthalmol..

[9] Clark G.M. (2015). The multi-channel cochlear implant: Multi-disciplinary development of electrical stimulation of the cochlea and the resulting clinical benefit. Hear. Res..

[10] Aziliz L., Emeline D., Christian B. (2018). A review on mechanical considerations for chronically-implanted neural probes. J. Neural Eng..

[11] Rousche P.J., Normann R.A. (1998). Chronic recording capability of the utah intracortical electrode array in cat sensory cortex. J. Neurosci. Methods.

[12] Barrese J.C., Aceros J., Donoghue J.P. (2016). Scanning electron microscopy of chronically implanted intracortical microelectrode arrays in non-human primates. J. Neural Eng..

[13] Barrese J.C., Rao N., Paroo K., Triebwasser C., Vargas-Irwin C., Franquemont L., Donoghue J.P. (2013). Failure mode analysis of silicon-based intracortical microelectrode arrays in non-human primates. J. Neural Eng..

[14] Griffith R.W., Humphrey D.R. (2006). Long-term gliosis around chronically implanted platinum electrodes in the rhesus macaque motor cortex. Neurosci. Lett..

[15] Kotov N.A., Winter J.O., Clements I.P., Jan E., Timko B.P., Campidelli S., Pathak S., Mazzatenta A., Lieber C.M., Prato M. (2009). Nanomaterials for neural interfaces. Adv. Mater..

[16] Prasad A., Xue Q.S., Sankar V., Nishida T., Shaw G., Streit W.J., Sanchez J.C. (2012). Comprehensive characterization and failure modes of tungsten microwire arrays in chronic neural implants. J. Neural Eng..

[17] Massey L.K. (2002). Permeability Properties of Plastics and Elastomers: A Guide to Packaging and Barrier Materials.

[18] Jones D. (1996). Principles and Prevention of Corrosion.

[19] Deiasi R., Russell J. (1971). Aqueous degradation of polyimides. J. Appl. Polym. Sci..

[20] Rubehn B., Stieglitz T. (2010). In vitro evaluation of the long-term stability of polyimide as a material for neural implants. Biomaterials.

[21] Polikov V.S., Tresco P.A., Reichert W.M. (2005). Response of brain tissue to chronically implanted neural electrodes. J. Neurosci. Methods.

[22] Nordhausen C.T., Maynard E.M., Normann R.A. (1996). Single unit recording capabilities of a 100 microelectrode array. Brain Res..

[23] Kipke D.R., Vetter R.J., Williams J.C., Hetke J.F. (2003). Silicon-substrate intracortical microelectrode arrays for long-term recording of neuronal spike activity in cerebral cortex. IEEE Trans. Neural Syst. Rehabil. Eng..

[24] Cogan S.F. (2008). Neural stimulation and recording electrodes. Annu. Rev. Biomed. Eng..

[25] Saddow S.E., Frewin C.L., Nezafati M., Oliveros A., Afroz S., Register J., Reyes M., Thomas S. 3C-SiC on Si: A bio- and hemo-compatible material for advanced nano-bio devices. Proceedings of the 2014 IEEE 9th Nanotechnology Materials and Devices Conference (NMDC).

[26] Frewin C.L., Locke C., Saddow S.E., Weeber E.J., Saddow S.E. (2011). Biocompatibility of sic for neurological applications. Silicon Carbide Biotechnology: A Biocompatible Semiconductor for Advanced Biomedical Devices and Applications.

[27] Frewin L.C., Coletti C., Register J.J., Nezafati M., Thomas S., Saddow E.S., Demarchi D., Tagliaferro A. (2015). Silicon carbide materials for biomedical applications. Carbon for Sensing Devices.

[28] Lei X., Kane S., Cogan S., Lorach H., Galambos L., Huie P., Mathieson K., Kamins T., Harris J., Palanker D. (2016). Sic protective coating for photovoltaic retinal prosthesis. J. Neural Eng..

[29] Saddow S.E., Frewin C., Reyes M., Register J., Nezafati M., Thomas S. (2014). 3C-SiC on Si: A biocompatible material for advanced bioelectronic devices. ECS Trans..

[30] Zorman C.A. Silicon carbide as a material for biomedical microsystems. Proceedings of the 2009 Symposium on Design, Test, Integration & Packaging of MEMS/MOEMS.

[31] Frewin C.L., Locke C., Mariusso L., Weeber E.J., Saddow S.E. Silicon carbide neural implants: In vivo neural tissue reaction. Proceedings of the 2013 6th International IEEE/EMBS Conference on Neural Engineering (NER).

[32] Cogan S.F., Edell D.J., Guzelian A.A., Ping Liu Y., Edell R. (2003). Plasma-enhanced chemical vapor deposited silicon carbide as an implantable dielectric coating. J. Biomed. Mater. Res. A.

[33] Deku F., Cohen Y., Joshi-Imre A., Kanneganti A., Gardner T.J., Cogan S.F. (2018). Amorphous silicon carbide ultramicroelectrode arrays for neural stimulation and recording. J. Neural Eng..

[34] Henry A., ul Hassan J., Bergman J.P., Hallin C., Janzén E. (2006). Thick silicon carbide homoepitaxial layers grown by cvd techniques. Chem. Vapor Depos..

[35] Hassan J., Bae H.T., Lilja L., Farkas I., Kim I., Stenberg P., Sun J.W., Kordina O., Bergman P., Ha S.Y., Janzén E. (2014). Fast growth rate epitaxy on 4° off-cut 4-inch diameter 4H-SiC wafers. Mater. Sci. Forum.

[36] Saxena V., Steckl A.J., Soo Park Y. (1998). Chapter 3 building blocks for sic devices: Ohmic contacts, schottky contacts, and p-n junctions. Semiconductors and Semimetals.

[37] Feng X., Huang Y., Rosakis A.J. (2007). On the stoney formula for a thin film/substrate system with nonuniform substrate thickness. J. Appl. Mech..

[38] Bernardin E., Frewin C.L., Dey A., Everly R., ul Hassan J., Janzén E., Pancrazio J., Saddow S.E. (2016). Development of an all-sic neuronal interface device. MRS Adv..

[39] Schroder D.K. (2006). Semiconductor material and device characterization. Carrier and Doping Density.

[40] Allongue P., Cachet H. (1985). Band-edge shift and surface-charges at illuminated n-GaAs aqueous electrolyte junctions. Surface state analysis and simulation of their occupation rate. J. Electrochem. Soc..

[41] Peter L.M., Giménez S., Bisquert J. (2016). Semiconductor electrochemistry. Photoelectrochemical Solar Fuel Production.

[42] Rose T.L., Robblee L.S. (1990). Electrical-stimulation with pt electrodes. VIII. Electrochemically safe charge injection limits with 0.2 ms pulses. IEEE Trans. Biomed. Eng..

[43] Peters D., Schorner R., Holzlein K.-H., Friedrichs P. (1997). Planar aluminum-implanted 1400 V 4h silicon carbide p-n diodes with low on resistance. Appl. Phys. Lett..

[44] Mitlehner H., Friedrichs P., Peters D., Schorner R., Weinert U., Weis B., Stephani D. Switching behaviour of fast high voltage sic pn-diodes. Proceedings of the 10th International Symposium on Power Semiconductor Devices and ICs (ISPSD’98).

[45] McConnell G.C., Rees H.D., Levey A.I., Gutekunst C.A., Gross R.E., Bellamkonda R.V. (2009). Implanted neural electrodes cause chronic, local inflammation that is correlated with local neurodegeneration. J. Neural Eng..

[46] Prasad A., Xue Q.-S., Dieme R., Sankar V., Mayrand R., Nishida T., Streit W., Sanchez J. (2014). Abiotic-biotic characterization of pt/ir microelectrode arrays in chronic implants. Front. Neuroeng..

[47] Simon D.M., Charkhkar H., St. John C., Rajendran S., Kang T., Reit R., Arreaga-Salas D., McHail D.G., Knaack G.L., Sloan A. (2017). Design and demonstration of an intracortical probe technology with tunable modulus. J. Biomed. Mater. Res. A.

[48] Reit R., Zamorano D., Parker S., Simon D., Lund B., Voit W., Ware T.H. (2015). Hydrolytically stable thiol-ene networks for flexible bioelectronics. ACS Appl. Mater. Interfaces.

[49] Teo A.J.T., Mishra A., Park I., Kim Y.-J., Park W.-T., Yoon Y.-J. (2016). Polymeric biomaterials for medical implants and devices. ACS Biomater. Sci. Eng..

[50] Kozai T.D.Y., Catt K., Li X., Gugel Z.V., Olafsson V.T., Vazquez A.L., Cui X.T. (2015). Mechanical failure modes of chronically implanted planar silicon-based neural probes for laminar recording. Biomaterials.

[51] Kip A.L., Jeffrey D.U., Junyan Y., David C.M., Daryl R.K. (2006). Chronic neural recordings using silicon microelectrode arrays electrochemically deposited with a poly(3,4-ethylenedioxythiophene) (pedot) film. J. Neural Eng..

[52] Wilks S., Richardson-Burn S., Hendricks J., Martin D., Otto K. (2009). Poly(3,4-ethylene dioxythiophene) (pedot) as a micro-neural interface material for electrostimulation. Front. Neuroeng..

[53] Aqrawe Z., Montgomery J., Travas-Sejdic J., Svirskis D. (2018). Conducting polymers for neuronal microelectrode array recording and stimulation. Sens. Actuat. B Chem..

[54] Block M.L., Hong J.S. (2005). Microglia and inflammation-mediated neurodegeneration: Multiple triggers with a common mechanism. Prog. Neurobiol..

[55] McHardy J., Geller D., Brummer S.B. (1977). An approach to corrosion control during electrical stimulation. Ann. Biomed. Eng..

[56] Cogan S.F., Ludwig K.A., Welle C.G., Takmakov P. (2016). Tissue damage thresholds during therapeutic electrical stimulation. J. Neural Eng..

[57] Do D.-H., Ecker M., Voit W.E. (2017). Characterization of a thiol-ene/acrylate-based polymer for neuroprosthetic implants. ACS Omega.

[58] Jones K.E., Campbell P.K., Normann R.A. Interelectrode isolation in a penetrating intracortical electrode array. Proceedings of the Twelfth Annual International Conference of the IEEE Engineering in Medicine and Biology Society.

[59] Jones K.E., Campbell P.K., Normann R.A. (1992). A glass/silicon composite intracortical electrode array. Ann. Biomed. Eng..

[60] Branner A., Stein R.B., Fernandez E., Aoyagi Y., Normann R.A. (2004). Long-term stimulation and recording with a penetrating microelectrode array in cat sciatic nerve. IEEE Trans. Biomed. Eng..

[61] Tanaka A. (2004). Toxicity of indium arsenide, gallium arsenide, and aluminium gallium arsenide. Toxicol. Appl. Pharmacol..

[62] Pakes A., Skeldon P., Thompson G.E., Fraser J.W., Moisa S., Sproule G.I., Graham M.J., Newcomb S.B. (2003). Anodic oxidation of gallium nitride. J. Mater. Sci..

[63] Kashiwada T., Watanabe T., Ootani Y., Tateyama Y., Einaga Y. (2016). A study on electrolytic corrosion of boron-doped diamond electrodes when decomposing organic compounds. ACS Appl. Mater. Interfaces.

[64] Pan M., Liang L., Lin W., Kim S.M., Li Q., Kong J., Dresselhaus M.S., Meunier V. (2016). Modification of the electronic properties of hexagonal boron-nitride in bn/graphene vertical heterostructures. 2D Mater..

[65] Saddow S.E. (2013). SiC biotechnology for advanced biomedical applications. Second Workshop on Advanced Cybernetics.

[66] Saddow S.E. (2011). Silicon Carbide Biotechnology: A Biocompatible Semiconductor for Advanced Biomedical Devices and Applications.

[67] Kordina O., Saddow S.E., Saddow S.E., Agarwal A. (2004). Silicon carbide overview. Advances In Silicon Carbide Processing and Applications.

[68] Frewin C.L., Oliveros A., Locke C., Filonova I., Rogers J., Weeber E., Saddow S.E. (2009). The development of silicon carbide based electrode devices for central nervous system biomedical implants. Mater. Res. Soc. Symp. Proc..

[69] Osburn C.M., Raider S.I. (1973). Effect of mobile sodium ions on field enhancement dielectric breakdown in SiO_2_ films on silicon. J. Electrochem. Soc..

[70] Jansz P., Hinckley S. Double boundary trench isolation effects on a stacked gradient homojunction photodiode array. Proceedings of the 2008 Conference on Optoelectronic and Microelectronic Materials and Devices.

[71] Meyer R.D., Cogan S.F., Nguyen T.H., Rauh R.D. (2001). Electrodeposited iridium oxide for neural stimulation and recording electrodes. IEEE Trans. Neural Syst. Rehabil. Eng..

[72] Cogan S.F., Ehrlich J., Plante T.D., Smirnov A., Shire D.B., Gingerich M., Rizzo J.F. (2009). Sputtered iridium oxide films for neural stimulation electrodes. J. Biomed. Mater. Res. Part B Appl. Biomater..

[73] Cogan S.F., Ehrlich J., Plante T.D., Gingerich M.D., Shire D.B. (2010). Contribution of oxygen reduction to charge injection on platinum and sputtered iridium oxide neural stimulation electrodes. IEEE Trans. Biomed. Eng..

[74] Randles J.E.B. (1947). Kinetics of rapid electrode reactions. Discuss. Faraday Soc..

[75] Stoner B.R., Raut A.S., Brown B., Parker C.B., Glass J.T. (2011). Graphenated carbon nanotubes for enhanced electrochemical double layer capacitor performance. Appl. Phys. Lett..

[76] Aryan N.P., Asad M.I.H.B., Brendler C., Kibbel S., Heusel G., Rothermel A. In vitro study of titanium nitride electrodes for neural stimulation. Proceedings of the 2011 Annual International Conference of the IEEE Engineering in Medicine and Biology Society.

[77] Meijs S., Alcaide M., Sørensen C., McDonald M., Sørensen S., Rechendorff K., Gerhardt A., Nesladek M., Rijkhoff N.J.M., Pennisi C.P. (2016). Biofouling resistance of boron-doped diamond neural stimulation electrodes is superior to titanium nitride electrodes in vivo. J. Neural Eng..

[78] Krishnan R. (2007). Fundamentals of semiconductor electrochemistry and photoelectrochemistry. Encyclopedia of Electrochemistry.

[79] Clarke R.A., Green M.A., Shewchun J. (1974). Contact area dependence of minority-carrier injection in schottky barrier diodes. J. Appl. Phys..

[80] Ilatikhameneh H., Ameen T., Chen F., Sahasrabudhe H., Klimeck G., Rahman R. (2018). Dramatic impact of dimensionality on the electrostatics of p-n junctions and its sensing and switching applications. IEEE Trans. Nanotechnol..

[81] Willis A.J. (1990). Edge effects in schottky diodes. Solid State Electron..

[82] Moon B.H., Han G.H., Kim H., Choi H., Bae J.J., Kim J., Jin Y., Jeong H.Y., Joo M.-K., Lee Y.H. (2017). Junction-structure-dependent schottky barrier inhomogeneity and device ideality of monolayer MoS_2_ field-effect transistors. ACS Appl. Mater. Interfaces.

[83] Neudeck P.G. (2000). Electrical impact of sic structural crystal defects on high electric field devices. Mater. Sci. Forum.

[84] McCreery D.B., Agnew W.F., Yuen T.G.H., Bullara L. (1990). Charge density and charge per phase as cofactors in neural injury induced by electrical stimulation. IEEE Trans. Biomed. Eng..

[85] Weiland J.D., Anderson D.J., Humayun M.S. (2002). In vitro electrical properties for iridium oxide versus titanium nitride stimulating electrodes. IEEE Trans. Biomed. Eng..

[86] Jan E., Hendricks J.L., Husaini V., Richardson-Burns S.M., Sereno A., Martin D.C., Kotov N.A. (2009). Layered carbon nanotube-polyelectrolyte electrodes outperform traditional neural interface materials. Nano Lett..

[87] Stuart F.C., Philip R.T., Julia E., Christina M.G., Timothy D.P. (2007). The influence of electrolyte composition on the in vitro charge-injection limits of activated iridium oxide (AIROF) stimulation electrodes. J. Neural Eng..

[88] Piret G., Hébert C., Mazellier J.-P., Rousseau L., Scorsone E., Cottance M., Lissorgues G., Heuschkel M.O., Picaud S., Bergonzo P. (2015). 3d-nanostructured boron-doped diamond for microelectrode array neural interfacing. Biomaterials.

[89] Pernot J., Volpe P.N., Omnès F., Muret P., Mortet V., Haenen K., Teraji T. (2010). Hall hole mobility in boron-doped homoepitaxial diamond. Phys. Rev. B.

[90] Vasilevskiy K.V., Roy S.K., Wood N., Horsfall A.B., Wright N.G. (2017). On electrons mobility in heavily nitrogen doped 4H-SiC. Mater. Sci. Forum.

[91] Bieber J.A., Saddow S.E., Moreno W.A. Synthesis of nanoscale structures in single crystal silicon carbide by electron beam lithography. Proceedings of the Fifth IEEE International Caracas Conference on Devices, Circuits and Systems.

[92] Rosenbloom A.J., Nie S., Ke Y., Devaty R.P., Choyke W.J. (2006). Columnar morphology of porous silicon carbide as a protein-permeable membrane for biosensors and other applications. Mater. Sci. Forum.

[93] Saddow S.E., Melnychuk G., Mynbaeva M., Nikitina I., Vetter W.M., Jin L., Dudley M., Shamsuzzoha M., Dmitriev V., Wood C.E.C. (2011). Structural characterization of sic epitaxial layers grown on porous sic substrates. MRS Proc..

