# Correction: Bernardin E.K.; et al. Demonstration of a Robust All-Silicon-Carbide Intracortical Neural Interface. *Micromachines*, 2018, *9*, 412

**DOI:** 10.3390/mi9090451

**Published:** 2018-09-10

**Authors:** Evans K. Bernardin, Christopher L. Frewin, Richard Everly, Jawad Ul Hassan, Stephen E. Saddow

**Affiliations:** 1 Department of Biomedical Engineering, University of South Florida, Tampa, FL 33620, USA; ebernardin@mail.usf.edu; 2 Department of Bioengineering, University of Texas at Dallas, Dallas, TX 75080, USA; Christopher.frewin@utdallas.edu; 3 Nanotechnology Research and Education Center @ USF, Tampa, FL 33617, USA; everly@usf.edu; 4 Department of Physics, Chemistry and Biology (IFM), Linköping University, SE-581 83 Linköping, Sweden; jawad.ul-hassan@liu.se; 5 Department of Electrical Engineering, University of South Florida, Tampa, FL 33620, USA

The authors would like to indicate the following financial support they received to the Acknowledgement Section of their published paper [1]: “The Florida Education Fund’s McKnight Doctoral Fellowship Program and the Alfred P. Sloan Foundation University Center of Exemplary Mentoring (UCEM) are gratefully acknowledged for providing financial support to Evans K. Bernardin during this research.”

The changes do not affect the scientific results. We apologize for any inconvenience caused to the readers. The manuscript will be updated and the original will remain online on the article webpage, with a reference to this Correction. 

## References

[1] Bernardin E.K., Frewin C.L., Everly R., Hassan J.U., Saddow S.E. (2018). Demonstration of a Robust All-Silicon-Carbide Intracortical Neural Interface. Micromachines.

